# Retreatment Strategies for Cases Containing Calcium Silicate-Based Root Canal Sealers: A Comprehensive Review

**DOI:** 10.3390/dj12020041

**Published:** 2024-02-18

**Authors:** Hussain Al akam, Hyeon-Cheol Kim, Ji Wook Jeong

**Affiliations:** 1 Department of Endodontics, School of Dentistry, The University of Texas Health Science Center at Houston, 7500 Cambridge Street, Suite 6400, Houston, TX 77054, USA; alakamhussain@gmail.com; 2 Department of Conservative Dentistry, Pusan National University School of Dentistry, Yangsan 50612, Republic of Korea; golddent@pusan.ac.kr

**Keywords:** calcium silicate-based root canal sealer, retreatment, solvent

## Abstract

This review explores the field of retreatment strategies for cases filled with calcium silicate-based root canal sealers. Since the introduction of calcium silicate-based materials in dentistry, calcium silicate-based root canal sealers have become popular among dentists because of their biocompatibility, bioactivity, and sealing ability. Therefore, effective retreatment strategies are indispensable. This article aims to identify the challenges associated with the removal of calcium silicate-based sealers themselves and removal of gutta-percha with the sealers during retreatment, evaluate current techniques and materials, and provide future directions for research in this field. Regarding the strategies of removal of root canal sealers, calcium silicate-based sealers are still relatively new materials for clinicians compared with traditional sealers such as epoxy- or eugenol-based sealers. First, no clinically established solvents have been reported. Second, calcium silicate-based sealers are currently utilized by clinicians in either the cold sealer-based technique or the warm vertical condensation technique. Third, the setting process of calcium silicate-based sealers generates byproducts, primarily calcium hydroxide and secondarily hydroxyapatite, that could interact with dentine. Lastly, there is a lack of clinical studies evaluating the efficacy of retreatment protocols for teeth filled with calcium silicate-based sealers. Therefore, it is important to investigate the chemo-mechanical properties of calcium silicate-based sealers themselves and their reactions to solvents and/or mechanical instruments and identify the interfacial properties of calcium silicate-based sealers with respect to dentine and gutta-percha. In addition, researchers in the clinical field need to actively gather and report data on retreatments of teeth filled with calcium silicate-based sealers.

## 1. Introduction

### 1.1. Calcium Silicate-Based Sealer (CSS)

A surge in the popularity of calcium silicate-based sealers (CSSs) has been noticed recently in the field of endodontics. Since iRoot SP™ (Innovative Bioceramics, Vancouver, Canada) was first introduced in 2007, many new brands of CSSs have been marketed, and more dentists are interested in using CSSs. The properties of CSSs are unique because of the setting process and their byproducts, which contribute to their popularity.

### 1.2. Properties of CSSs

The unique properties of CSSs include their setting process and their byproducts. CCSs are hydraulic sealers that require the presence of water for their setting. The setting process includes the reaction of the CSS with water. This reaction results in the formation of calcium hydroxide as a byproduct [1].

2(3CaO·SiO_2_) + 6H_2_O = 3CaO·2SiO_2_·3H_2_O + 3Ca(OH)_2_

2(2CaO·SiO_2_) + 4H_2_O = 3CaO·2SiO_2_·3H_2_O+Ca(OH)_2_

The set sealer produced has been found to be biocompatible and bioactive [2,3]. Hydroxyapatite, as a secondary byproduct, is deposited on the surface of the set sealer, contributing to the bioactivity of the set CSS [3]. The emanation of calcium hydroxide ions from CSSs elevates the pH, which contributes to the sealers’ antimicrobial efficacy [2,4].

### 1.3. Outcome of Root Canal Treatment Using CSSs

The outcomes of non-surgical initial root canal treatments and retreatments using CSSs encourages more clinicians to use CSSs for obturation. In a non-randomized clinical trial, a calcium silicate-based sealer in combination with the single-cone technique demonstrated success rates, ranging from 84% to 90%, while the epoxy resin-based sealer with a continuous wave condensation technique showed success rates from 80% to 89% [5]. In addition, a randomized clinical trial compared the success rate of a CSS with the single-cone technique and an epoxy resin-based sealer with the continuous wave condensation technique. The results showed an average success rate of 94.3% for the CSS with the single-cone technique in comparison to 92.3% for the teeth obturated with the epoxy resin-based sealer using the continuous wave condensation technique [6]. These promising results and favorable outcomes encourage and motivate clinicians to use CSSs more extensively in the practice of endodontics. The increased use of CSSs among endodontists and general practitioners has prompted the need to implement new strategies of retreatment for cases obturated with CSSs. For instance, regaining patency is a potential challenge that can occur during the removal of CSSs [7].

## 2. Retreatment

### 2.1. Reason for Retreatment

In general, the success of endodontic treatment can be evaluated by the absence of signs and symptoms of infection or inflammation, including pain, tenderness to palpation and percussion, and the absence of any soft tissue signs of infection, such as swelling, in combination with radiographic evidence of periapical lesion healing and bone formation and regaining the mastication force and normal function of the treated teeth [8,9]. The presence of signs and/or symptoms is the main reason for retreatment. The purpose of retreatment is to eliminate the presence of persistent infection in the root canal system. Radiographic presence of periapical pathology is an important diagnostic method that has been used to evaluate healing after root canal treatments [10,11,12]. Reinfection can occur because of various factors, such as poor coronal restoration or the presence of missed root canals [13,14,15]. The presence of radiographic radiolucency before the initial endodontic treatment has been found to increase the possibility of failure and the need for retreatment as a result [16]. In addition, retreatment using modern technology has been found to be beneficial to patients’ quality of life [9].

### 2.2. Factors Affecting the Outcome of Retreatment

**Figure 1:**
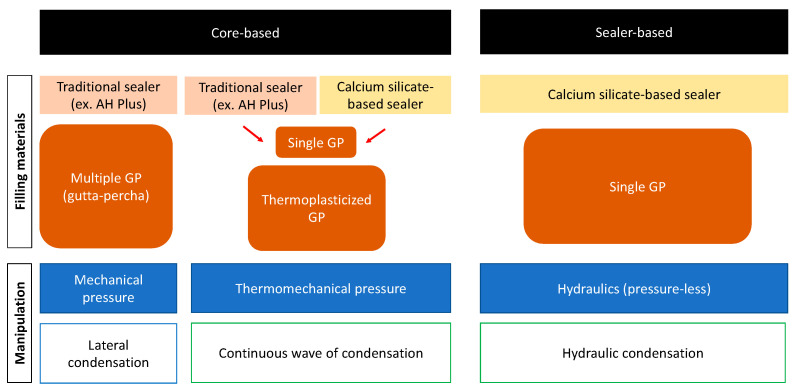
Schematic concepts of obturation methods.

Numerous studies have discussed the outcome of endodontic retreatment. Endodontic retreatment showed high success rates in most of these studies. Ng et al. [17], in their prospective study, which included annual clinical and radiographic evaluation of teeth which had been initially treated and retreated, found that the success rate of retreatment was comparable to that of the initial treatment. The success of retreatment is influenced by many factors. These factors include the presence of periapical radiolucency and its size [17], case selection [18,19], accessibility to the obturation material throughout coronal restoration [20], and retrievability of the different obturation materials [21]. The last factor is critical for clinicians to expect a favorable prognosis before executing the retreatments of teeth obturated with CSSs due to the absence of solvents. Friedman et al. [21] divided the obturation materials based on their setting process into soft-setting pastes, which are easy to remove and clean, and hard-setting cements, which might require the usage of solvents in combination with mechanical debridement to remove [21]. In addition to the obturation material, the obturation technique is another critical factor in determining the complicity of retreatment (Figure 1). DeLong et al. [22] compared the push-out bond strength of two CSSs (MTA Plus Sealer™ (Avalon Biomed Inc, Bradenton, FL, USA) and EndoSequence BC Sealer™ (Brasseler USA, Savannah, GA, USA)) and an epoxy resin-based sealer, AH Plus™ (Dentsply De Trey Gmbh, Konstanz, Germany), using both the single-cone obturation technique and the continuous wave obturation technique. They found that the continuous wave technique decreased the bond strength of the MTA Plus Sealer™ and that the CSS with a single cone demonstrated the highest push-out resistance [22]. This study showed that the obturation technique might influence the setting properties of the sealers. Athkuri et al. [23] assessed the retrievability of root canal filling material obturated with cold lateral condensation, warm vertical condensation, and thermoplasticized injectable techniques along with the AH Plus™ sealer and BioRoot RCS™ (Septodont, Saint-Maur-des-Fossés, France). The samples that were obturated with the thermoplasticized technique demonstrated a higher percentage of root filling residuals after the removal of root fillings than the lateral condensation or warm vertical compaction techniques. However, there were no significant relationships between the type of sealer used and the amount of residual filling [23]. This indicates that the obturation technique used during the initial endodontic treatment affects the retrievability of the root filling material during retreatment. Therefore, clinically, the outcome of retreatment is affected by multiple factors, and its outcome varies depending on the various scenarios.

### 2.3. Challenges and Risks Specific to CSSs

No solvents have decisively proven to be effective in dissolving CSSs. For this reason, the challenges associated with the retrievability of CSSs might depend on the final setting of the sealers. Soft-setting CSSs are easier to remove from the root canal system, whereas hard-setting CSSs might necessitate the use of solvents in combination with mechanical debridement [21,22].

## 3. Literature Review

### 3.1. Literature Search

Two reviewers (J.J., H.A.) conducted a comprehensive literature search from the 1 June 2020 to the 1 December 2023 to identify studies related to the topic in the PubMed and Google Scholar databases. The following search keywords were used to find relevant studies: (calcium silicate-based sealers Physicochemical and Biological properties) OR (calcium silicate-based sealers retreatment) OR (effect of obturation technique on retrievability of calcium silicate-based sealers) OR (use of calcium silicate-based sealers in endodontic procedure) OR (outcome of root canal treatment and retreatment) OR (Relationship between periapical lesions and root canal treatment outcome) AND (modern and contemporary calcium silicate-based sealers retreatment methods). Laboratory and clinical studies investigating the properties of CSSs and the retreatment of teeth obturated with CSSs were included. Studies performed on resin teeth or animal teeth were excluded.

### 3.2. Calcium Silicate-Based Sealers Properties in Relation to Retreatment

The properties of calcium silicate-based sealers allow clinicians to use either a sealer-based technique or warm vertical condensation. Mann et al. [2] studied the physicochemical and biological properties of EndoSequence BC Sealer HiFlow™ (CSS) (Brasseler USA, Savannah, GA, USA) and compared them with those of EndoSequence BC Sealer™ (CSS) and AH Plus sealer™ (resin epoxy-based sealer). At room temperature, both CSSs had a similar flow, which was lower than the flow of the AH Plus™ sealer. However, upon increasing the temperature to 100 °C, the flow of EndoSequence BC Sealer HiFlow™ was found to be the highest, followed by AH Plus™ and EndoSequence BC Sealer™. Interestingly, increasing the temperature to 150 °C put the flow of AH Plus™ ahead of the flow of EndoSequence BC Sealer HiFlow™ and EndoSequence BC Sealer™. Regarding solubility, both CSSs showed a similar solubility, which was significantly higher than the solubility of AH Plus™. No difference was found among the three sealers regarding their antibacterial effect, whereas both CSSs demonstrated a higher biocompatibility than AH Plus [2]. These results indicate that it is necessary to thoroughly remove CSSs from the root canal systems in retreatment because of the flowability of CSSs, while a CSS is more soluble than an epoxy resin-based sealer. The capacity of adhesion between CSSs and dentine has been the scope of many studies. Resistance to dislodgement measures the capacity of adhesion between sealers and dentine. Sagsen et al. [24] compared the push-out resistance of I Root SP™, a calcium silicate-based sealer, MTA Fillapex™, a salicylate resin- and calcium silicate-based sealer, and AH Plus™, an epoxy resin-based sealer (Table 1). They found that I Root SP™ has a similar push-out bond resistance to AH Plus™, while MTA Fillapex™ has the lowest among the three sealers [24]. In a similar study, Donnermeyer et al. [25] compared three CSSs (Total Fill BC Sealer™ (FKG, La Chaux-de-Fonds, Switzerland), Endo CPM Sealer™ (Egeo, Buenos Aires, Argentina), and BioRoot RCS™ (Septodont, St. Maur-des-Fossés, France)) with an epoxy resin-based sealer (AH Plus™) (Table 1). The study revealed that the dislodgment resistance of AH Plus™ was significantly higher than that of all three CSSs [25]. The CSSs in these studies demonstrated different values of dislodgement resistance, while some types of CSSs were comparable to the push-out resistance of epoxy resin-based sealers.

### 3.3. Adhesion and Interfacial Space between CSS and Gutta-Percha

Research on the adhesive capacity of CSSs has predominantly focused on their bonding force to dentine, with less attention given to their bonding capacity with gutta-percha. It has been claimed by commercial manufacturers that gutta-percha cones treated with calcium silica particles have better and stronger adhesion to CSSs than conventional gutta-percha. An SEM study compared the adhesion of TotalFill BC sealer™ (Roeko, Langenau, Germany) and AH Plus sealer™ to conventional gutta-percha and BC gutta-percha using single-cone and lateral condensation techniques. The study did not find a significant difference in the voids between the two types of gutta-percha and the two types of sealers regardless of the obturation technique [26]. The current literature on the adhesion capacity of CSSs to gutta-percha is insufficient, and further investigation is needed. In an ex vivo micro CT study, De-Deus et al. [27] showed that the frequency of the presence of gaps was more common between a single gutta-percha cone and CSSs than between a single-cone gutta-percha and AH Plus and theorized that the hydrophobic nature of gutta-percha cone repulses CSSs due to their hydrophilic nature.

### 3.4. Potential Changes in Interfacial Dentin by CSSs

The impact of CSSs on interfacial dentine’s microstructure is another topic of interest. Atmeh et al. [28] evaluated the effect of Biodentine™ (calcium silicate-based restorative material) on dentine using confocal laser scanning microscopy, scanning electron microscopy, micro-Raman spectroscopy, and two-photon auto-fluorescence and second-harmonic generation imaging. It was found that the interaction between dentine and Biodentine™ forms a “mineral infiltration zone” (MIZ) because of the mineral precipitates within the dentinal tubules that result from the hydration of the calcium silicate-based material. These products lead to the degradation of the collagen component of the interfacial dentin. This leads to the movement of ions in the dentine and increases the mineralization in this region of the dentine [28]. Jeong et al. [29] also observed the formation of an MIZ only in samples incubated for 14 days after obturation and not in those incubated for 3 days only, suggesting a time-dependent process [29]. This leads to the conclusion that the formation of an MIZ is the result of an extensive interaction that starts after the sealer is set. Despite the previous reports which investigated the presence of the MIZ, its formation, and its chemical composition, there is currently insufficient evidence regarding its effect on endodontic treatments.

### 3.5. Different Scenarios in Retreatment

While there is general agreement among clinicians that retreatment should cause the minimum change to the root canal’s anatomy, concerns are still present regarding the regain of apical patency in cases obturated with CSSs, especially those cases where gutta-percha does not reach the working length. In an ex vivo study, Hess et al. [30] created an experimental model in which they compared the possibility of regaining patency in samples obturated with AH Plus sealer™ and EndoSequence BC sealer™. The teeth samples were divided into four groups. In two of the groups, the samples were obturated with a gutta-percha that reached the working length, whereas, in the other two groups, the samples were obturated with a gutta-percha that was 2 mm shorter than the working length. Heat, chloroform, rotary instruments, and hand files were used in the retreatment protocol. The success rate of regaining working length in samples where the gutta-percha reached the working length was 100%, regardless of the sealer used. In the samples where the gutta-percha was 2 mm shorter than the working length, the success rate was 100% for the samples obturated with AH Plus™ in comparison with 30% in the samples obturated with EndoSequence BC sealer™. Patency was regained in 100% of the samples in both of the groups obturated with AH Plus™, while it was regained in 80% of the samples in the group obturated with EndoSequence BC sealer™ and gutta-percha reaching the working length. Patency was regained in 30% of the samples that had been obturated with EndoSequence BC sealer™ and gutta-percha 2 mm shorter than the working length [30]. In contrast, a recent ex vivo study compared the potential of regaining patency in samples obturated with EndoSequence BC sealer™ in which gutta-percha was placed 1.5 mm shorter than the working length using 10% formic acid, 20% hydrochloric acid, and chloroform; patency was regained in 100% of the samples retreated with 10% formic acid and 20% hydrochloric acid, a figure which was not significantly different for the samples in which chloroform had been used (93%) [31]. This discrepancy in results between different studies can be attributed to the lack of standardization in preparing the samples rather than to the effect of the solvents or the techniques used. This highlights the importance of standardizing sample design in research on this topic.

### 3.6. Different Nature of CSSs

The physical strength of the set CSS is a critical factor that determines its retrievability. Different brands of CSSs have different degrees of retrievability, mainly because of their different setting properties. Soft-set sealers are easier to retrieve compared to hard-set sealers [32]. Carillo et al. [32] compared the possibility of regaining patency in canals that had been obturated with three CSS: EndoSequence BC sealer™, EdgeBioceramic™ (EDGEENDO, Albuquerque, NM, USA), and NeoSEALERFlo™ (Avalon Biomed, Houston, TX, USA). It was shown that canals filled with NeoSEALERFlo™ have a higher success rate in regaining patency than canals filled with the others. The authors found that re-establishing patency could be affected by which CSS was used and that NeoSEALERFlo™ might be labelled as a soft-setting CSS. Therefore, the presence of hard-setting CSSs might lead to more difficulties and complications during the retreatment procedure. Some challenges that can be faced include the separation of instruments, perforation, and difficulty in reaching the proper working length [21].

### 3.7. Removal of CSSs

Regaining patency in cases obturated with CSS during retreatment is one of the major prognostic factors for positive outcomes. The presence of CSSs apical to the gutta-percha in cases where the gutta-percha did not reach the working length can demonstrate a real obstacle during retreatment [30].

In addition, the presence of CSSs in areas of the root canal system that are inaccessible to mechanical means of debridement might form another obstacle preventing the complete removal of CSSs in endodontic retreatment procedures. An ex vivo study that evaluated the removal of root canal filling material from the mesial canals of mandibular molars connected with an isthmus using the XP-endo Finisher R instrument with or without solvent concluded, after comparing the micro CT scans of the samples before and after the treatment, that the use of solvent did not improve the clearance of the filling materials from the canals or isthmuses, whereas mechanical debridement using the XP-endo Finisher R instrument facilitated the removal of the filling materials but could not remove them completely [33]. Horvath et al. [34] used SEM and photographs in an ex vivo study to assess the efficacy of two solvents, chloroform, and eucalyptol, in removing the filling gutta-percha and sealers from root canal walls and dentinal tubules. More remnants of gutta-percha and sealers on the canal walls and inside the dentinal tubules were found in groups where solvents had been used compared with groups without solvents. It was suggested that more root canal filling material was pushed inside the dentinal tubules because of the dissolution of the root canal material by the solvents, which led to the compacting of the root canal filling material inside the dentinal tubules [34]. Donnermeyer et al. [35] compared the retrievability of three calcium silicate-based sealers to AH Plus™ from round and patent root canals using mechanical instrumentation. They found that the complete removal of sealers was not achievable even in round and patent root canals, with the percentage of remaining sealers ranging from 2.1% to 28.2% for all four sealers in all the groups of the study [35]. The study showed that the complete removal of CSSs from the dentine wall was not achieved regardless of the simple anatomy of the canals.

## 4. Current Retreatment Techniques

Current CSSs retrieval techniques can be divided into chemical and mechanical techniques.

### 4.1. Solvents for Gutta-Percha

The gutta-percha cone is a semisolid obturation material. It is considered the material of choice in modern endodontics. Excessive mechanical removal of the gutta-percha can lead to alterations in the anatomy of the root canals. Solvents are commonly recommended to avoid any alteration in the anatomy and facilitate the removal of the gutta-percha. Chloroform is the most effective solvent used for the removal of gutta-percha. Its properties of fast action, strength, and fast evaporation make it a respectable choice as a solvent. However, chloroform has since been found to be cytotoxic and carcinogenic; therefore, extrusion beyond the apex should be avoided. The use of xylene and eucalyptol as alternatives to chloroform has been suggested, but they have also been found to be less effective and impractical to use clinically. Wennberg and Ørstavik suggested the use of methyl chloroform as an effective, less toxic alternative to chloroform. It has been found to be less effective than chloroform but more effective than xylene and eucalyptol [36,37].

### 4.2. Chemical Dissolution of CSSs

The possible chemicals that can be employed in the retrieval of CSSs have been evaluated in many studies. Numerous studies have examined the difference in solubility between CSSs and epoxy resin-based sealers and the effect of different solvents on CSSs. Borges et al. [38] subjected AH Plus™, iRoot SP™, MTA Fillapex™, Sealapex™ (Sybron Endo/Kerr Co, Orange, CA, USA), and MTA-Angelus™ (Angelus, Londrina, PR, Brazil) to solubility tests to compare their surface structure changes and ion release (Table 2). The samples of the sealers were assessed using scanning electron microscopy and energy-dispersive spectroscopy. The study found that iRoot™, MTA Fillapex™, and Sealapex™ have a higher solubility than AH Plus™ and MTA-A™. The study also revealed that CSSs exhibit a high release of calcium ions [38]. The pH of the surrounding environment was shown to be a critical factor in the solubility of CSSs (Table 2). Endo Sequence BC Sealer™ was found to be significantly more soluble than AH Plus™ in a low pH [39]. This implies that acids might have the potential to serve as solvents for CSSs. Thermal treatment as a physical means was found to be effective in altering the structure of CSSs and impacting their solubility [40]. Ideal solvents are intended to only affect sealers without affecting the integrity of dentine. Garrib et al. [41] studied the effect of irrigating with 17% EDTA along with either 10% or 20% formic acid on the integrity of TotalFill BC sealer™ and the integrity of dentine (Table 3). The study found that irrigating with 17% EDTA and 10% formic acid did not affect the integrity of the dentine. However, irrigation affected the integrity of the CSS used in the study and aided its mechanical removal. The use of 20% formic acid was found to corrode the integrity of dentine [41]. The efficacy of 20% hydrochloric acid and chloroform in comparison with 10% formic acid in regaining apical potency has been investigated. There is no significant difference among them in terms of regaining patency [31]. Therefore, 10% formic acid is deemed to be a promising solvent when used to penetrate CSSs and regain patency.

### 4.3. Mechanical Removal of CSSs

The use of conventional hand files and modern rotary and NiTi files in the removal of CSSs has been assessed in multiple studies. Donnermeyer et al. [35] compared the efficacy and retreatment time of Hedström files, Reciproc R40, Mtwo retreatment file R 25/0.06, Mtwo 40.06, and F6 SkyTaper size 040 in the removal of CSSs and epoxy resin-based sealers (Table 3). Regarding the amount of sealer remnants, all NiTi rotary files performed much better than the Hedström hand files, regardless of the sealer type. The F6 SkyTaper instruments were found to be the fastest when compared with all the files that were being assessed [35]. Furthermore, the use of reciprocating files occupied a portion of the interest in the mechanical techniques for removing CSSs. Kırıcı et al. [42] compared two reciprocal systems, M-Wire Reciproc and Reciproc Blue, and evaluated their efficacy in the removal of CSSs and their competence in preserving the root canal’s anatomy in curved canals using micro-CT. No significant difference in the amount of residual was found between the two systems. However, apical canal transportation was found to be significantly higher in the M-Wire Reciproc group, but the formation of incomplete and complete cracks was witnessed with both systems [42]. Despite the positive results that were reached in these past studies with different mechanical means for the removal of CSSs, no mechanical technique was found to completely remove CSSs from the root canal system.

### 4.4. Integrating Technology into Retreatment of CSSs

The use of modern mechanical techniques has been discussed in various studies. Fruchi et al. [43] conducted a micro-CT evaluation to measure the efficacy of sealer removal using the Reciproc R25 instrument or WaveOne Primary files along with xylene and passive ultrasonic irrigation in curved canals (Table 3). The Reciproc instrument and the WaveOne Primary files showed a removal efficacy of 93% and 92%, respectively. The use of xylene and PUI helped in the removal of the root canal filling material but did not increase the percentage of the removed filling material significantly [43]. Wright et al. compared the usage of two modern irrigation protocols, EndoVac and GentleWave, to the efficacy of the usage of a side-vented needle in the removal of root canal filling materials. Micro-CT imaging was used to evaluate the percentage of the removal of the root canal filling material for all three irrigation techniques. GentleWave removed the highest ratio of the residuals of the root canal filling material, followed by the side-vented needle. EndoVac was found to remove the least amount of residuals of the root canal filling material [44]. Wright and Fruchi [43,44] found that AH Plus was more difficult to remove than CSSs (Table 3). The efficacy of shock wave-enhanced emission photoacoustic streaming (SWEEPS) in CSS removal has also been evaluated. Angerame et al. [45] compared the efficacy of SWEEPS with the efficacy of passive ultrasonic irrigation (PUI) in the removal of CSSs ex vivo (Table 3). The combination of reciprocating instrumentation with SWEEPS provided more satisfying results than the usage of reciprocating instrumentation combined with PUI [45]. The XP-endo Finisher R system removed more CSSs compared to the ultrasonic-assisted irrigation or EndoActivator [46]. Despite continuous effort to assess the efficacy of mechanical techniques and modern irrigation technology in the removal of CSSs, no technique has been proved to completely remove CSSs from the root canal system in the literature (Table 3).

**Table 2:** Sealers used in the solubility studies mentioned in this review.

Sealers	Manufacturers	Composition	Solubility (%)	Reference
iRoot SP	Innovative BioCeramix Inc., Vancouver, Canada	Zirconium oxide, calcium silicates, calcium phosphate, calcium hydroxide filler, and thickening agents	20.64 ± 1.42	[38]
MTA Fillapex	Angelus, Londrina, PR, Brazil	Components after mixture: resins (salicylate, diluting, natural), radiopaque bismuth, nanoparticulated silica, mineral trioxide aggregate, and pigments	14.89 ± 0.73	[38]
Sealapex	Sybron Endo/Kerr Co, Orange, CA, USA	Calcium oxide, bismuth trioxide, zinc oxide, submicron silica, titanium dioxide, zinc stearate, tricalcium phosphate, ethyl toluene sulphonamide, poly(methylene methyl salicylate) resin, isobutyl salicylate, and pigments	5.65 ± 0.80	[38]
MTA-A	Angelus, Londrina, PR, Brazil	Tricalcium silicate, dicalcium silicate, tricalcium aluminate, tetracalcium aluminoferrite, bismuth oxide, iron oxide, calcium carbonate, magnesium oxide, crystalline silica, and residues (calcium oxide, free magnesium oxide, potassium, and sodium sulphate compounds)	−1.24 ± 0.19	[38]
AH Plus	Dentsply De Trey Gmbh, Konstanz, Germany	Component A: epoxy resin, calcium tungstate, zirconium oxide, aerosil, and iron oxide. Component B: adamantane amine, N,N-Dibenzyl-5-oxanonane, TCD-Diamine, calcium tungstate, zirconium oxide, and aerosi	0.28 ± 0.08	[38]
AH Plus Jet	Dentsply DeTrey Gmbh, Konstanz, Germany	Bisphenol A/F epoxy resin, calcium tungstate, zirconium oxide, silica, iron oxide pigments dibenzyldiamine, aminoadamantane, and silicone oil	0.04 ± 0.11 to 0.26 ± 0.15 ^2^	[39]
EndoSequence BC Sealer	Brasseler USA, Savannah, GA, USA	Zirconium oxide, calcium silicates, calcium phosphate monobasic, calcium hydroxide filler, and thickening agents	4.96 ± 0.94 to 12.88 ± 0.94 ^2^	[39]

^2^ The study demonstrated a difference in the solubility related to the time and the pH. The difference in solubility was demonstrated with a range of values.

**Table 3:** Materials and instruments used in the treatment studies.

Name	Company	Type	Reference
XP-endo Finisher R instrument	FKG Dentaire, La Chaux-de-Fonds, Switzerland	Mechanical	[33]
Eucalyptol	Biodinamica, Ibiporã, PR, Brazil	Chemical	[33]
Reciproc R40 NiTi files	VDW, Munich, Germany	Mechanical	[35]
Mtwo retreatment file R 25/0.06	VDW GmbH, Munich, Germany	Mechanical	[35]
F6 SkyTaper size 040	KOMET, Lemgo, Germany	Mechanical	[35]
10% formic acid	Sigma Aldrich, Gillingham, UK	Chemical	[41]
20% formic acid	Sigma Aldrich, Gillingham, UK	Chemical	[41]
ProTaper Gold finisher file	Dentsply, Charlotte, USA	Mechanical	[41]
M-Wire Reciproc	VDW GmbH, Munich, Germany	Mechanical	[42]
Reciproc Blue	VDW GmbH, Munich, Germany	Mechanical	[42]
xylene	Not mentioned	Chemical	[43]
Reciproc R25 instrument	VDW, Munich, Germany	Mechanical	[43]
WaveOne Primary instrument	Dentsply Maillefer, Tulsa, USA	Mechanical	[43]
Reciproc Blue files	VDW GmbH, Munich, Germany	Mechanical	[45]
XP-endo Finisher R system	XPR; FKG Dentaire, La Chaux-de-Fonds, Switzerland	Mechanical	[46]

### 4.5. Clinical Studies

Clinical studies describing the outcome of retreatment obturated with CSSs are limited in number compared to those of traditional sealers. CSSs are still relatively new materials to be used in endodontics, and further examination and assessment are needed.

## 5. Future Directions

Regarding the retrievability of CSSs, current evidence is still insufficient for guiding clinicians in their decision making. Therefore, researchers in the clinic field need to develop more standardized protocols for clinical studies evaluating the effectiveness of CSSs in retreatment.

### 5.1. Advancement of Technology

The employment of modern techniques and technology has been assessed, as mentioned before, in various studies, e.g., ultrasonic-assisted irrigation, EndoActivator, passive ultrasonic irrigation, SWEEPS, and GentleWave. Some of these technologies demonstrate promising data. More studies are needed to explore their potential and describe their limitations. 

### 5.2. Research Gaps

Many research gaps were found during the preparation of this paper. This is in addition to the lack of standardization in measuring the retrievability of CSSs. For example, both Hess et al. and Rezaei et al. used a similar study design, but the distance of the short obturation was not standardized and was different between the two studies [30,31]. In addition, in similar study designs, the absence of voids in the sealer in the apical portion should be verified using the available modern technology. Such standardization would help in obtaining more accurate related results. The nature of CSSs’ setting should be the next point of focus. Carillio et al. [32] investigated the possibility of regaining patency, but the hardness of the set sealer was not investigated. We suggest that CSSs should be categorized into soft- and hard-setting sealers depending on their setting process and properties, and each group should be studied extensively to reach a better understanding regarding their nature and properties and the clinical reflection of these properties. The effect of solvents on dentine, the cells surrounding the root, and CSSs should be studied more extensively using standardized methods. Cutting-edge analytical methods to measure the solubility of sealers and the effect of solvents on dentine would provide a deeper insight into the strategies of retreatment.

## 6. Conclusions

The complete retrievability of CSSs from the root canal system using the current strategies and techniques has not yet been achieved. The persistent challenges of regaining patency and removing CSSs from spaces that are inaccessible to instruments continue to be evident. The integration of mechanical and chemical removal techniques, combined with supplementary irrigation methods, is yielding promising outcomes in CSS retrieval. More standardized studies are needed regarding the nature of all available CSSs and the effectiveness of modern technology in their retrievability. 

## Figures and Tables

**Table 1:** Sealers used in the push-out bond studies that were mentioned in this review.

Sealers	Manufacturers	Push-Out Force (N/mm^2^)	References
I Root SP	Innovative BioCreamix Inc., Vancouver, Canada	1.52 to 2.6 ^1^	[24]
MTA Fillapex	Angelus Solucxoes Odontologicas, Londrina, Brazil	0.6 to 1.37 ^1^	[24]
AH Plus	Dentsply DeTrey GmbH, Konstanz, Germany	1.9 to 2.9 ^1^	[24]
BioRoot RCS	Septodont, Saint-Maur-des-Fossés, France	1.96 to 2.76 ^1^	[25]
Endo C.P.M. Sealer	EGEO, Buenos Aires, Argentina	1.47 to 1.82 ^1^	[25]
Total Fill BC Sealer	FKG, La Chaux-de-Fonds, Switzerland	2.95 to 3.89 ^1^	[25]
AH Plus	Dentsply, Konstanz, Germany	6.12 to 8.62 ^1^	[25]

^1^ A difference in push-out bond strength was demonstrated, depending on the corono-apical position of the tested tooth’s sections. The difference in push-out bond strength was demonstrated with a range of values.
